# Polymer Blends of Polyetherimide and Poly(ether ester urethane): Controlling Dielectric Properties for Ultrahigh Energy Storage

**DOI:** 10.3390/polym17233100

**Published:** 2025-11-22

**Authors:** Tao Lu, Shiqi Wang, Jinfeng Li, Tian Zhang

**Affiliations:** 1 Electronic Information School, Wuhan University, Wuhan 430072, China; taolu9@whu.edu.cn (T.L.); 2023302121262@whu.edu.cn (S.W.); 2 Suzhou Institute of Wuhan University, Suzhou 215000, China

**Keywords:** dielectrics, energy storage, high temperature, blending strategy, hydrogen bonding interactions

## Abstract

Polymer dielectrics offer outstanding advantages for high-power energy storage applications, such as high breakdown strength (*E_b_*) and efficiency (*η*), while both decrease rapidly at elevated temperature (>150 °C). Although several strategies including nanocomposites and crosslinking have been evaluated to enhance *E_b_* and heat resistance, the discharged energy density (*U_d_*) of polymer dielectrics is still limited by the low dielectric constant (*K*). Herein, we have implemented a blending strategy by utilizing hydrogen bonding interactions between molecular chains for polyetherimide (PEI) and poly(ester ether urethane) (PEEU). Both the experimental and computational simulation results reveal that the blending can contribute to the increased molecular chain spacing and control the charge transport by destroying the conjugated structure to broaden bandgap and induce deep traps, improving the *K* and *E_b_* simultaneously. As a result, the blend film achieves an unprecedented *U_d_* of 5.50 with the *η* above 90% at 200 °C. Furthermore, it exhibits stable performances during ultralong 10^5^ charge–discharge cycles in harsh environments (250 MV/m and 200 °C). This work opens a new avenue to scalable high Ud all-polymer dielectric for high-temperature applications and promotes the understanding of the dielectric behavior of polymer blend films.

## 1. Introduction

The urgent demand for reliable energy storage in high-temperature applications, such as electric vehicles and aerospace systems, requires dielectric capacitors that can function stably above 140 °C [1,2,3,4,5]. Currently, commercial polymer dielectrics like biaxially oriented polypropylene (BOPP) are limited to maximum operating temperatures below 100 °C [6,7,8,9]. This thermal limitation highlights the pressing need to develop advanced polymer dielectrics with superior reliability and thermal stability for high-temperature applications [10,11]. Polyetherimide (PEI) stands out as a promising candidate due to its high glass transition temperature (*T_g_*) and high breakdown strength (*E_b_*), along with its cost-effectiveness and scalability [12]. However, a significant challenge persists: the charge-discharge efficiency (*η*) and discharged energy density (*U_d_*) of PEI-based polymers significantly decline at elevated temperatures and high electric fields [13,14,15]. This performance drop is mainly due to a sharp increase in conduction current, underscoring the need for further material optimization to fully harness its potential in demanding environments [16,17,18].

The key to addressing the issues of high-temperature capacitors based on polyetherimide (PEI) is to suppress the conduction current effectively [19]. Theoretically, the conduction mechanism in conjugated polymers, such as PEI, primarily arises from the delocalization of *π* electrons within and between polymer chains, which is fundamentally influenced by the material’s conformation and morphology [20,21,22]. Specifically, the planar backbone structure of PEI enhances intra-chain electron delocalization and the *π*-conjugation effect, which significantly undermines energy storage performance [23,24,25]. Therefore, decreasing the planarity of the polymer chain is considered an effective strategy. This modification can be achieved through interface engineering, which involves increasing chain spacing and introducing non-covalent interactions, such as hydrogen bonds [19,26]. However, while this strategy effectively blocks conduction current, it often compromises the polymer’s inherent high thermal stability or mechanical strength, presenting a major challenge in achieving a comprehensive balance of performance [27,28,29].

This study outlines a strategy for developing an all-organic blend system by cleverly choosing poly(arylene ether urea) (PEEU) and polyetherimide (PEI) as blend components. PEEU’s high inherent polarity is due to the high dipole moment (4.56 D) associated with its polar urea groups, which contributes to a higher intrinsic dielectric constant (*K* = 4.7) compared to conventional polyureas [6]. Critically, as a high *T_g_* semicrystalline polymer, these same urea units serve as deep charge traps, essential for suppressing high-field conduction losses at elevated temperatures. Furthermore, the semi-crystalline nature of PEEU makes its structure highly sensitive to blending and processing, allowing for the targeted tuning of dielectric performance [30]. Unlike traditional inorganic filler composite systems, this approach offers two significant advantages. First, the physical entanglement of molecular chains significantly enhances PEEU’s film-forming processability, addressing its tendency to become brittle when formed into a film alone [31]. Second, the gradient matching of dielectric parameters between the components effectively suppresses interfacial space charge accumulation, thereby synergistically boosting both the breakdown strength (*E_b_*) and energy storage efficiency (*η*) of the composite system. The critical factor is the precise regulation of dielectric behavior through hydrogen bond-induced interface engineering. The formation of hydrogen bonds disrupts the planarity of the PEI main chain, breaks the delocalization and conjugate structure of *π* electrons, and introduces deep-level traps at the interface. This significantly increases the potential barrier for charge injection and migration, thereby substantially suppressing conduction current at high temperatures and ensuring high efficiency. Performance tests indicate that by increasing the molecular chain spacing and optimizing structural polarization in the blend system, the dielectric constant (*K*) is effectively enhanced while maintaining a high *E_b_*, reaching 6.51 at the optimal ratio. The blend film ultimately reaches an exceptional energy storage density (*U_d_*) of 10.74 J/cm^3^ at room temperature, maintaining a high efficiency (*η*) of 91.57%. This study not only introduces a novel approach for the scalable production of high-energy-storage all-polymer dielectrics suitable for high-temperature applications but also enhances the understanding of the mechanisms underlying dielectric behavior in polymer blend films.

## 2. Materials and Methods

### 2.1. Materials and Characterization

All materials used, detailed characterization methods, and specific simulation procedures are fully described in the Supplementary Materials.

### 2.2. Preparation of Polymer Films

**Figure 1:**
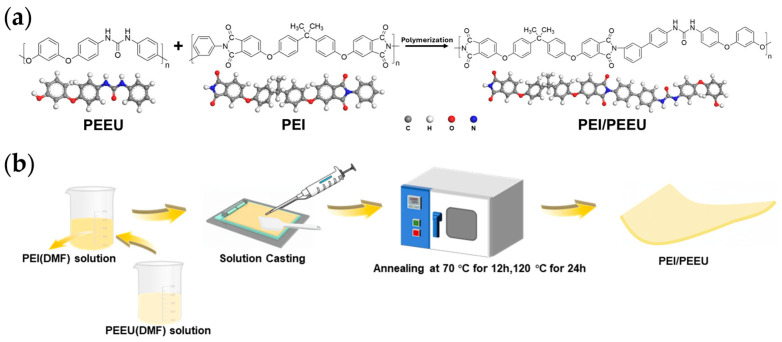
(**a**) The polymerization process of PEI and PEEU, (**b**) Preparation of PEI/PEEU films.

First, PEEU powder and PEI powder were each dissolved in DMF solvent at a concentration of 2 wt.%. Each mixture was stirred at 1500 revolutions per minute at 50 °C for 24 h until fully dissolved, forming separate PEEU and PEI solutions. These solutions were then combined according to the required mass ratio and stirred continuously at 1500 revolutions per minute at 50 °C for 6 h to ensure uniform mixing. The resulting solution was applied to a clean glass plate and evenly spread using a doctor blade, with the film thickness maintained between 7 μm and 12 μm. The coated film was then placed in an oven for staged drying: initially at 70 °C for 12 h to remove most of the solvent, followed by 24 h at 120 °C to ensure complete solvent evaporation. After drying, the film and glass plate were immersed in deionized water to detach the film, which was carefully removed to prevent damage. Finally, the peeled film was placed in a vacuum oven and dried at 50 °C for 6 h to eliminate any remaining moisture and solvent, resulting in a dry PEI/PEEU mixed film. This film was then stored in a desiccator for future use. The synthesis process of PEI/PEEU is illustrated in Figure 1a. Figure 1b presents a simplified flowchart of the film preparation process. Initially, each material is dissolved separately before being mixed, which enhances the control over the dispersion uniformity. This method ultimately contributes to improved performance consistency of the film (Figure S1) [6,30].

## 3. Results and Discussion

### 3.1. Structure Characterization

**Figure 2:**
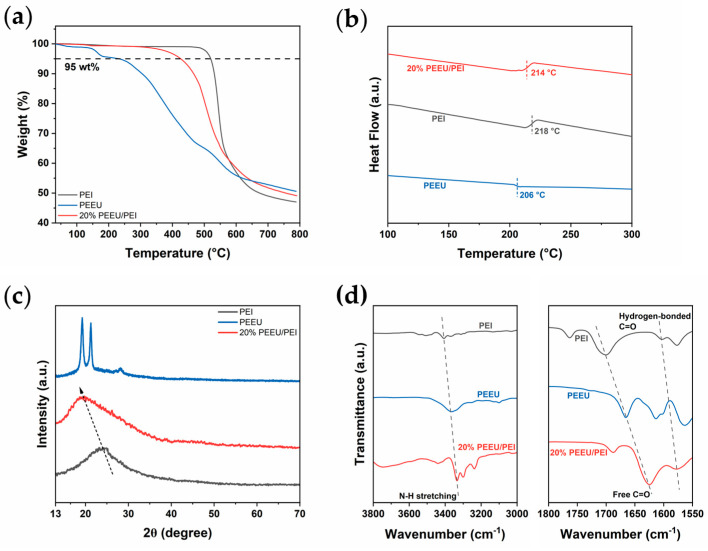
(**a**) TGA curves, (**b**) DSC curves, (**c**) XRD plots (The arrow indicates the increase in interlayer spacing.) and (**d**) FT-IR.

The thermal stability of the composite system was carefully assessed. Thermogravimetric analysis (TGA) confirmed that the polyetherimide (PEI) matrix primarily determined the thermal resistance, and the 20% PEEU/PEI blend exhibited a higher 5% mass loss temperature (Figure 2a), significantly surpassing the target operating temperature. Differential scanning calorimetry (DSC) results indicate that although the addition of PEEU (*T_g_* = 206 °C) slightly lowered the *T_g_* of pure PEI from 218 °C to 214 °C in the blend (Figure 2b), this value still far exceeded the 200 °C service temperature requirement. These results show that 20% PEEU/PEI composites have the necessary thermal stability and structural strength to ensure reliable long-term operation in high-temperature dielectric applications.

Scanning electron microscopy (SEM) was employed to characterize the structural morphology of PEI and 20% PEEU/PEI (Figures S2 and S3), which strongly confirms the excellent miscibility and successful blending of PEEU within the PEI matrix. Further characterizations include X-ray diffraction (XRD) and Fourier transform infrared (FT-IR) spectroscopy. XRD patterns confirm that both the PEI base material and PEI/PEEU composite systems primarily exhibit an amorphous structure (Figure 2c), indicated by a broad, diffuse diffraction peak centered around 18° to 20°. Significantly, the addition of PEEU does not induce significant crystallization in the PEI matrix; instead, it causes a slight shift of the amorphous peak toward lower 2θ angles. This shift implies an increase in interchain distance within the polymer structure, consistent with the introduction of new components that subtly disrupt the original chain packing. Such structural changes are often desirable for enhancing dielectric properties by facilitating chain mobility and potentially reducing the efficiency of charge hopping pathways [32,33]. FT-IR analysis conclusively demonstrates the presence of intermolecular hydrogen bonds between PEEU and PEI chains, which is fundamental to this blending strategy (Figure 2d) [34]. In the N-H stretching vibration region, pure PEEU exhibits a characteristic absorption peak. Upon the 20% PEEU/PEI curve, this peak significantly red shifts to lower wavenumbers within the 3300–3500 cm^−1^ range, with an increase in intensity. This shift confirms the formation of a stronger hydrogen-bonding network involving the N-H groups in PEEU. Additionally, in the C=O stretching vibration region, the characteristic peak of free C=O groups shifts downward after blending within the 1650–1750 cm^−1^ range. The emergence of new or enhanced shoulder peaks in the low-wavenumber region aligns with the formation of hydrogen-bonded C=O species. These spectral changes collectively confirm the successful formation of effective intermolecular hydrogen bonds between the PEEU and PEI components, leading to improvements in morphology and properties [16,35].

### 3.2. Dielectric Properties

**Figure 3:**
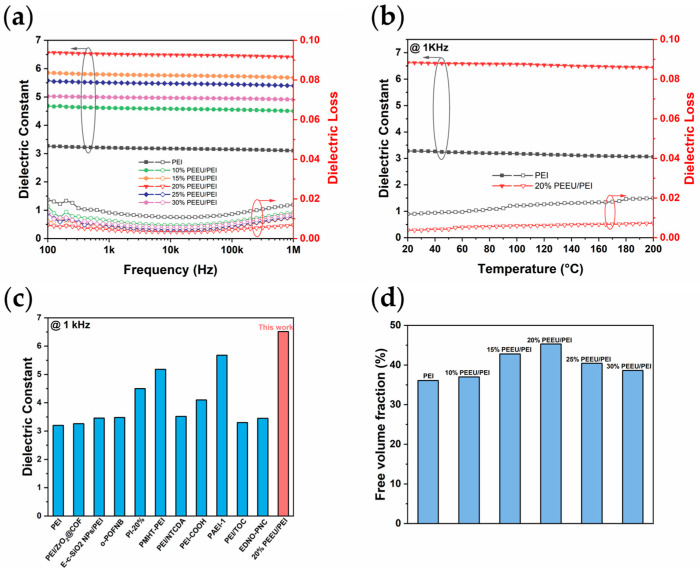
(**a**) Frequency-dependent dielectric constant and dielectric loss, (**b**) Temperature-dependent dielectric constant and dielectric loss, (**c**) Comparison of dielectric constant at 1 kHz and (**d**) Free volume fraction (FVF) diagram. The arrow and circle show the vertical axis corresponding to different curves.

The dielectric properties of PEEU/PEI blends, as a function of frequency and temperature, exhibit a significant enhancement. As shown in the frequency-dependent dielectric spectra (Figure 3a and Figure S4), the dielectric constant (*K*) of all blend systems remains stable over a broad frequency range (100 Hz to 1 MHz). Among them, the optimal 20% PEEU/PEI system shows a dielectric constant of 6.51 and a dielectric loss of 0.628% at 1 kHz, values that are significantly superior to those of pure PEI. The temperature-dependent dielectric spectra further confirm the excellent thermal stability of the blend. At 1 kHz, the *K* of the 20% PEEU/PEI blend fluctuates minimally, and the dielectric loss remains below 1% even at a high temperature of 200 °C (Figure 3b). This stability offers excellent advantages for high-temperature applications. Moreover, a comparison with other reported polymer dielectrics clearly shows that this study demonstrates a superior dielectric constant, fully proving the outstanding efficiency of this blending strategy (Figure 3c) [36,37,38,39,40,41,42,43,44,45].

The observed enhancement in the dielectric constant is structurally verified by the change in the chain packing. The loosening of molecular chain packing, which leads to an increase in the free volume fraction (FVF), is clearly indicated by the shift of the amorphous peak to a lower 2θ angle in the XRD pattern. The chain spacing of the polymer significantly increases and reaches a peak in the 20% PEEU/PEI blend (Figure 3d, Figures S5 and S6 and Table S2). This structural change is attributed to the disruption of the close packing of the planar PEI chains, primarily induced by the hydrogen bonding between PEEU and PEI. This loosening of chain packing promotes greater dipole polarization by allowing enhanced molecular segment mobility, thereby effectively increasing the dielectric constant (*K*) [46]. This principle, linking expanded interchain spacing to enhanced dielectric response due to reduced constraints, is well-established in polymer blend systems [47,48]. Therefore, the excellent dielectric properties are a direct result of the structural optimization achieved by PEEU blending modification, which effectively regulates the free volume and dipole response of the polymer.

**Figure 4:**
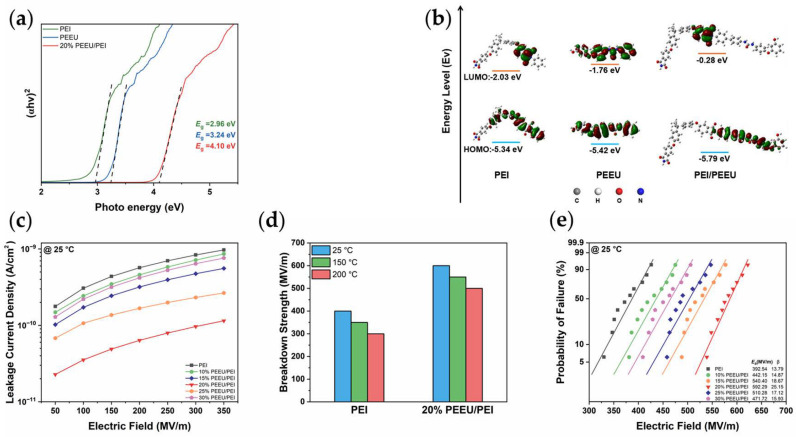
(**a**) UV−vis spectroscopy, (**b**) Energy level distribution, (**c**) Leakage current density curves at 25 °C, (**d**) Breakdown strength of PEI and 20% PEEU/PEI at 25 °C, 150 °C and 200 °C; (**e**) Weibull breakdown strength at 25 °C.

To further illustrate the impact of introducing PEEU on dielectric properties, ultraviolet-visible (UV-vis) spectroscopy clearly demonstrates a significant broadening of the optical band gap (*E_g_*) in the blend system (Figure 4a). Specifically, the calculated *E_g_* for the optimized 20% PEEU/PEI blend is approximately 4.10 eV, a notable improvement over the 2.96 eV of pure PEI. This finding is corroborated by density functional theory (DFT) calculations, which show a similar trend at the molecular level (Figure 4b). The introduction of PEEU effectively disrupts the π-conjugated system of the PEI backbone, significantly increasing the energy gap between the highest occupied molecular orbital (HOMO) and the lowest unoccupied molecular orbital (LUMO). This is consistent with the relatively weak electrostatic interactions observed in the blended molecules, as shown in Figure S7 [49,50,51]. This enlarged band gap is crucial for creating a deeper potential barrier, preventing electron injection and transport, and effectively suppressing the migration of charge carriers along the molecular chain.

Structural and electronic modifications have directly enhanced the insulation performance of PEI/PEEU. The leakage current density curves show that PEI/PEEU blends effectively suppress leakage current over a wide range of temperatures and electric fields (Figure 4c). At 25 °C, the leakage current density of the 20% PEEU/PEI blend is an order of magnitude lower than that of pure PEI. This suppression remains effective even at elevated temperatures (150 °C and 200 °C) (Figure S8), addressing the critical challenge of high conduction current in PEI-based materials. The significant decrease in leakage current density results from bandgap expansion and the formation of deep traps, which effectively restrict the movement of thermally excited carriers [8,52].

The PEEU blending strategy, as a definitive measure of electrical robustness, has significantly enhanced the breakdown strength (*E_b_*). The electric polarization versus electric field (*P–E*) loops of mixtures with different ratios are shown in Figures S9–S15. Figure 4d illustrates that the *E_b_* of the 20% PEEU/PEI composite markedly surpasses that of pure PEI across all tested temperatures. Notably, the 20% PEEU/PEI composite achieves an impressive *E_b_* of approximately 600 MV/m at room temperature. The Weibull probability plot further confirms this increased reliability (Figure 4e and Figure S16), indicating a significant shift in the failure distribution of the blended system toward a higher electric field. Evidence of bandgap widening, sharp suppression of leakage current, and a substantial increase in breakdown strength highlight the practical value of the PEEU blending strategy for developing reliable high-temperature dielectric capacitors.

### 3.3. High-Temperature Energy Storage Capabilities

**Figure 5:**
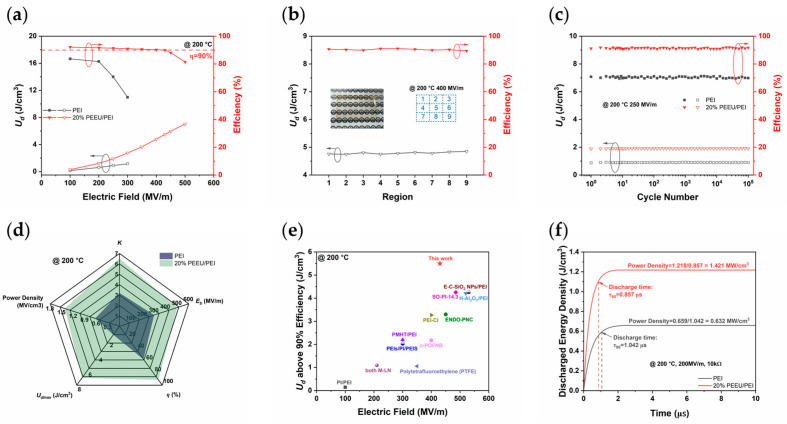
(**a**) *U_d_* and efficiency of PEI and 20% PEEU/PEI at 200 °C, (**b**) *U_d_* and efficiency of 20% PEEU/PEI dielectric films measured at nine regions under 200 °C and 400 MV/m, the inset is the photograph of 20% PEEU/PEI film, (**c**) Cyclic performance at 200 °C and 250 MV/m, (**d**) The radar plot, (**e**) Comparison of the *U_d_* above 90% efficiency of 20% PEEU/PEI and recently published high-temperature dielectric polymers, (**f**) Fast discharge curves of PEI and 20% PEEU/PEI at 200 °C. The arrow and circle show the vertical axis corresponding to different curves.

The superior electrical properties directly translate into exceptional energy storage performance, even under extreme thermal conditions. The *U_d_* and efficiency (*η*) curves across various temperatures reveal that the blend system significantly outperforms the pure PEI matrix (Figure 5a and Figure S17). While the *U_d_* of pure PEI rapidly declines above 200 MV/m at 200 °C, the 20% PEEU/PEI blend achieves a high *U_d_* of approximately 5.50 J/cm^3^ at 430 MV/m and maintains an impressive *η* exceeding 90%. This accomplishment is noteworthy for all-polymer dielectrics operating at such temperatures and electric fields. Furthermore, area reliability tests assessing the *U_d_* across nine different regions of the film confirm that the 20% PEEU/PEI blend exhibits excellent uniformity and structural integrity, ensuring dependable performance in large-area devices (Figure 5b, Figures S18 and S19).

The blend not only demonstrates high *U_d_* and uniformity but also exhibits exceptional long-term reliability. Cyclic performance data (Figure 5c and Figure S20) reveal that the 20% PEEU/PEI film maintains a stable *U_d_* of approximately 1.91 J/cm^3^ even after 10^5^ charge-discharge cycles at 200 °C and 250 MV/m, with *η* exceeding 90%. This remarkable operational stability indicates that PEEU modification effectively mitigates degradation mechanisms under repeated thermal and electrical stress. The radar chart offers a comprehensive overview, further highlighting the excellent energy storage performance of PEI/PEEU at elevated temperatures (Figure 5d). Notably, when compared to previously reported polymer materials, the 20% PEEU/PEI blend achieves an unmatched level of performance, reaching the highest *U_d_* at a lower electric field strength while maintaining high efficiency at 150 °C and 200 °C (Figure S21 and Figure 5e) [7,16,20,26,36,37,38,39,40,41,42,43,44,45,53,54,55,56,57,58,59,60]. This places the work at the leading edge of high-temperature polymer dielectric research.

The power density characteristics demonstrate the rapid energy transfer ability of the blend. The power density of the 20% PEEU/PEI composite is significantly enhanced, reaching 1.421 MW/cm^3^, along with a fast discharge time (*τ*_90_, defined as the time required to discharge 90% of the total stored energy), of only 0.857 μs. This performance far exceeds that of PEI, which has a power density of only 0.632 MW/cm^3^, attributed to its optimized molecular structure, which enables rapid charge-discharge. These comprehensive results establish that this work presents a promising and scalable dielectric candidate material for next generation high-temperature power electronic devices.

## 4. Conclusions

In summary, we have successfully demonstrated an efficient all-polymer blending strategy using PEEU and PEI to develop high-performance dielectric capacitors suitable for harsh environments. Central to this achievement is PEEU-induced interface engineering, which employs intermolecular hydrogen bonds to adjust both the structural and electronic properties of the polymer matrix. This structural disruption increases the free volume fraction and significantly broadens the electronic bandgap, effectively suppressing conduction currents across the entire temperature range. Consequently, the optimal 20% PEEU/PEI composite delivers unprecedented energy storage performance for all-polymer systems at high temperatures, achieving a discharge energy density (*U_d_*) of 5.50 J/cm^3^ at 200 °C and 430 MV/m, with efficiency (*η*) exceeding 90%. Furthermore, the blend exhibits excellent reliability over 10^5^ cycles and enhanced power density. This work not only introduces a new scalable and environmentally friendly approach to designing high *U_d_*, robust, and reliable dielectrics for applications such as electric vehicles and aerospace systems, but also demonstrates how noncovalent interactions can effectively regulate charge transport in blend films, providing key design principles for developing the next generation of high-performance polymer dielectrics.

## Data Availability

The original contributions presented in this study are included in the article/Supplementary Material. Further inquiries can be directed to the corresponding authors.

## Supplementary Materials

The following supporting information can be downloaded at: https://www.mdpi.com/article/10.3390/polym17233100/s1, Figure S1: Photograph of the polymer film (a) PEI film, (b) and (c) 20% PEEU/PEI film; Figure S2: SEM images of (a) PEI, (b) 20% PEEU/PEI; Figure S3: SEM images after cycling of (a) PEI, (b) 20% PEEU/PEI; Figure S4: Dielectric properties under different ratios of PEEU; Figure S5: The stereochemical structures of PEI and PEI/PEEU films by DFT calculation; Figure S6: Free volume simulation; Figure S7: Three-dimensional electrostatic potential; Figure S8: Leakage current density of PEI and 20% PEEU/PEI, (a) 150 °C, (b) 200 °C; Figure S9: Schematic diagram of *P-E* loop; Figure S10: *P-E* loops of (a) PEI, (b) 10% PEEU/PEI, (c) 15% PEEU/PEI, (d) 20% PEEU/PEI, (e) 25% PEEU/PEI, (f) 30% PEEU/PEI, at 25 °C; Figure S11: Comparison of *P-E* loop at 25 °C; Figure S12: *P-E* loops of (a) PEI, (b) 20% PEEU/PEI at 150 °C; Figure S13: Comparison of *P-E* loop at 150 °C; Figure S14: *P-E* loops of (a) PEI, (b) 20% PEEU/PEI at 200 °C; Figure S15: Comparison of *P-E* loop at 200 °C; Figure S16: Weibull breakdown strength of PEI and 20% PEEU/PEI (a) 150 °C, (b) 200 °C; Figure S17: Comparison of discharged energy density and charge-discharge efficiency of PEI and 20% PEEU/PEI, (a) 25 °C, (b) 150 °C; Figure S18: Energy storage performances of different regions in large scale 20% PEEU/PEI. The inset is the photograph of the 20% PEEU/PEI; Figure S19: Energy storage and dielectric constant performances of different regions in large scale 20% PEEU/PEI. Standard deviations of *U_d_* is 0.036. Standard deviations of *K* is 0.070; Figure S20: Cyclic performance at 150 °C, 250 MV/m; Figure S21: Maximum discharged energy density achieved above 90% efficiency at 150 °C of 20% PEEU/PEI and recently published high-temperature dielectric polymers; Table S1. Dielectric metrics of PEI and PEEU/PEIs; Table S2. Dielectric constant, Free volume fraction and Free Volume of PEI and PEEU/PEIs. References [7,8,20,26,36,37,38,41,53,54,55,56,61,62,63] are cited in the Supplementary Materials.
