# Supplementary material for: Polymer Blends of Polyetherimide and Poly(ether ester urethane): Controlling Dielectric Properties for Ultrahigh Energy Storage
Source: Polymers (Basel). 2025 Nov 22;17(23):3100. doi: 10.3390/polym17233100 (PMC12694172; doi:10.3390/polym17233100)
Supplement: Supplementary file 1 [file polymers-17-03100-s001.zip › polymers-3950743-supplementary.pdf]

Supplementary Materials for

Polymer Blends of Polyetherimide and Poly(ether ester urethane): Controlling Dielectric Properties for Ultrahigh Energy Storage

Tao Lu [1,2], Shiqi Wang [1,2], Jinfeng Li [1,2,*] and Tian Zhang [1,2,*]

[1] Electronic Information School, Wuhan University, Wuhan 430072, China; taolu9@whu.edu.cn (T.L.); 2023302121262@whu.edu.cn (S.W.)
[2] Suzhou Institute of Wuhan University, Suzhou 215000, China
* Correspondence: 2025102120035@whu.edu.cn (J.L.); txz908@whu.edu.cn (T.Z.)

Materials and methods

Materials

N,N-Dimethylformamide (DMF, 99.8%+, HPLC, Sigma-Aldrich) was sourced from Adamas (Shanghai, China). Polyetherimide (PEI, $M_n \approx 39{,}000$) and poly(ether ester urethane) (PEEU, $M_n \approx 30{,}000$) were supplied by Aladdin Reagent Co., Ltd. (Shanghai, China).

Electrical measurements

Unless otherwise specified, all samples measured for electrical properties were sputtered with gold electrodes for 90 seconds, achieving a typical thickness of 25 nm using the EMS Q150T ES Coater. Dielectric spectroscopy was conducted over a broad temperature range using a multi-frequency LCR meter (Keysight, E4980AL). Data were recorded at frequencies ranging from 100 Hz to 1 MHz, with temperatures spanning from 25 °C to 200 °C at a heating rate of 1 °C/min. The diameters of the upper and bottom

electrodes were set at 2 mm and 3 mm, respectively, resulting in an effective electrode area of 0.0314 cm^2. The thickness was maintained at approximately 7-12 μm.

The *P-E* loops were captured at a frequency of 100 Hz using a PolyK system for polarization and dielectric breakdown tests. The samples were placed in silicone oil with high-temperature resistance. Temperature control was achieved with a digital hot plate and a thermal couple. The breakdown strength was assessed with a Trek 20/20B high-voltage amplifier, applying a voltage ramp of 500 V per second. To determine the dielectric breakdown strength (E_b), a minimum of 10 points from various sample regions were chosen, and the breakdown strength of each point was assessed. The analysis of the results employed the two-parameter Weibull statistic formula: $P(E)=1-\exp[-(E/E_b)^{\beta}]$, where P(E) represents the cumulative probability of electric failure, E denotes the measured breakdown field of individual points, E_b signifies the electric field at which there is a 63.2% probability of sample breakdown (Weibull breakdown strength), and the shape parameter (β) characterizes the data scatter.

The thickness of the was controlled at about 5-10 μm. Leakage currents in the samples were measured with a Keithley 2410 electrometer and a Matsusada 10 kV high voltage source, under an electric field gradient of 50 MV/m. The upper electrode had a diameter of 2 mm, while the bottom electrode had a diameter of 3 mm, resulting in an effective electrode area of 0.0314 cm^2. The thickness of the sample was maintained at approximately 7-12 μm.

Structural Characterization

FTIR spectra of the films were obtained using a Tensor II FTIR spectrometer (BRUKER Company, Germany) in the 4000–500 cm^{-1} range at a resolution of 2.0 cm^{-1}. SEM analysis was performed with a HITACHIS-4800 super-resolution field emission scanning electron microscope. UV-vis spectra were measured with a Shimadzu UV-3600 (Japan), and the band gap was determined using the line cutting method correlating wavenumber and Abs.

XRD patterns of the films were obtained using a Rigaku-Dmax 2500 diffractometer, scanning angles (2 Theta) from 5° to 90°. Cu Kα radiation (λ = 1.54 Å) was employed. The molecular chain spacing was calculated using the Bragg equation, $2d\sin\theta = n\lambda$, where n is an integer multiple of the wavelength, d represents the chain distance, λ is the incident wave length, and θ is half of the scanning angle. DSC measurements were carried out on a TA Instruments Q2000 under a nitrogen atmosphere, with a heating/cooling rate of 10 °C min^{-1} over the range of 30 to 300 °C. TGA of the films was performed using a TA SDT Q600 under a nitrogen atmosphere (N_2) at a heating rate of 10 °C /min.

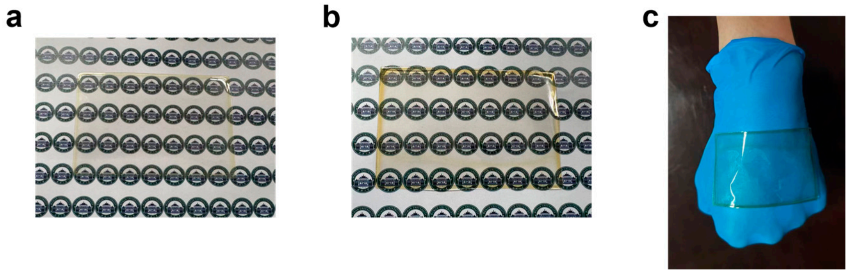

Figure S1. Photograph of the polymer film **(a)** PEI film, **(b)** and **(c)** 20% PEEU/PEI film.

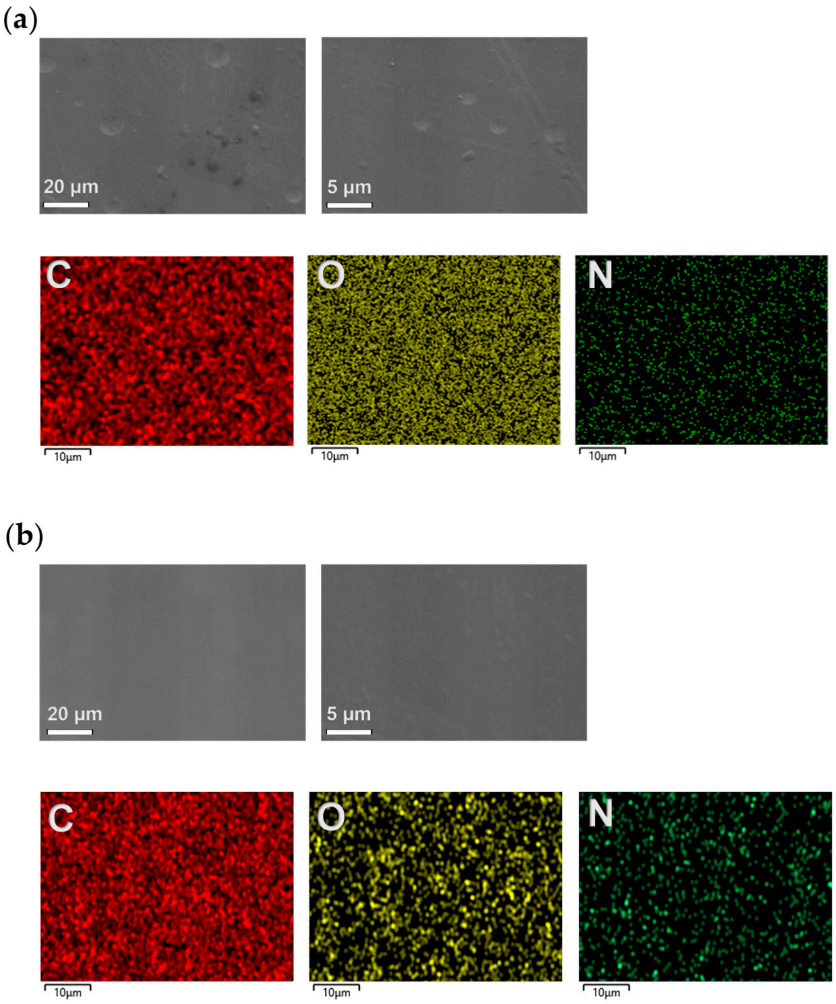

Figure S2. SEM images of **(a)** PEI, **(b)** 20% PEEU/PEI.

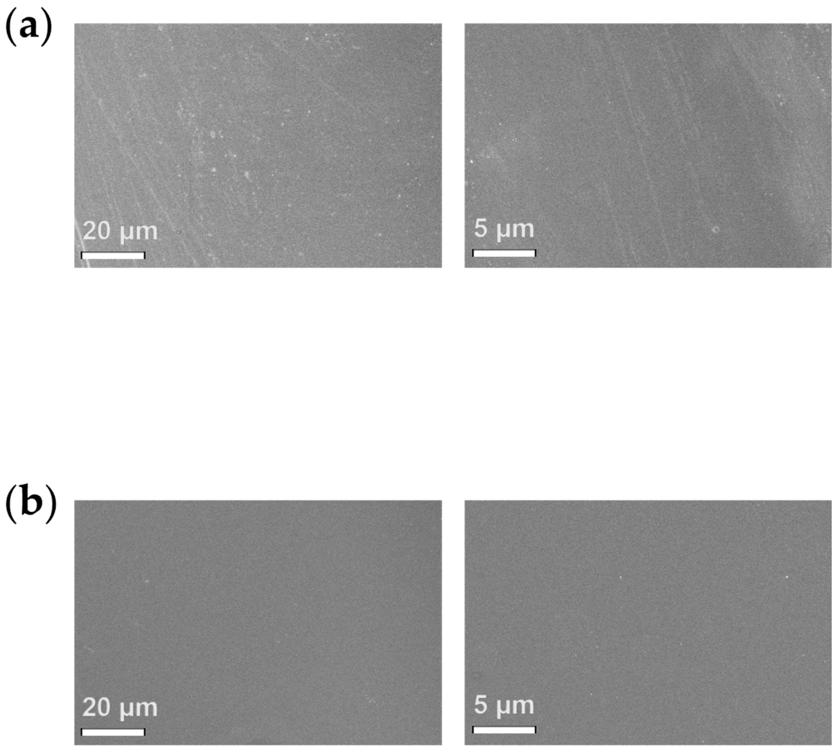

Figure S3. SEM images after cycling of **(a)** PEI, **(b)** 20% PEEU/PEI.

Density functional theory (DFT) calculation

Three-dimensional electrostatic potential

The optimal structure of polymer molecules must first be adjusted to achieve the most stable conformation with minimal energy in gas or liquid phases [1]. This stable conformation is then used as the initial structure for single-point energy calculations, utilizing a 6-31 G(d) basis set and B3LYP functional for more accurate molecular state wave function derivation. Subsequently, the molecular surface electrostatic potential, distribution of energy levels, π–π noncovalent interactions, and hydrogen bond interactions are visualized using GaussView, Multiwfn 3.8, and VMD.

Modeling construction

To mimic the self-adjusting nature of the multisite bonding network in polymers,

models were developed for PEI and PEEU/PEI. To enhance simulation efficiency, a chain length of 4 and a repeating unit of 10 were selected for the PEI model, yielding a simulation box size of 33.2 × 33.2 × 33.2 $Å^3$. The dimensions for the PEI/PEEU system were established as 36.7 × 36.7 × 36.7 $Å^3$.

Molecular dynamics simulation

Create an amorphous cell with the Amorphous Cell module. Start by optimizing the geometric structure and modeling the thermal imidization process with the COMPASS II function in the Geometry Optimization module of the Forcite Calculation. Next, conduct brief molecular dynamics simulations at a consistent temperature in the NVT ensemble, then proceed with NVE simulations to maintain constant energy. Lastly, determine the cohesive energy of the generated structures using GGA and BLYP functional methods as computational parameters.

Free volume analysis was performed on equilibrated molecular models (PEI, HPP-25%, and HSBN-1.5%) derived from molecular dynamics (MD) simulations. Fractional free volume visualization utilized a probe-based technique, involving a 1.0 Å radius spherical probe traversing the accessible amorphous structure volume. The simulation box was grid-discretized, segregating unoccupied volume into gray (occupied) and blue (free) based on probe accessibility [2]. This geometric assessment offers a quantitative and visual depiction of internal void architecture, crucial for linking structural attributes to gas transport and molecular mobility characteristics.

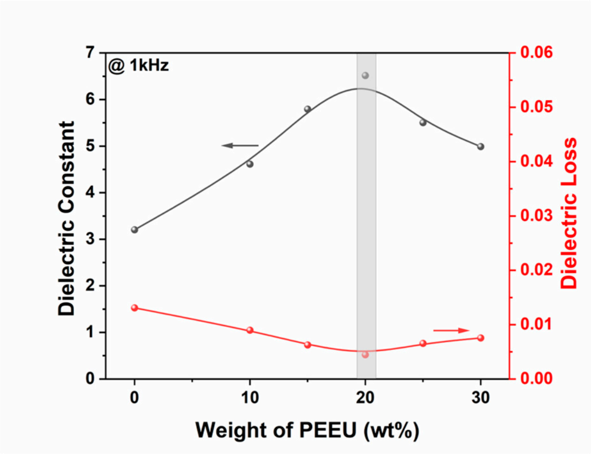

Figure S4. Dielectric properties under different ratios of PEEU.

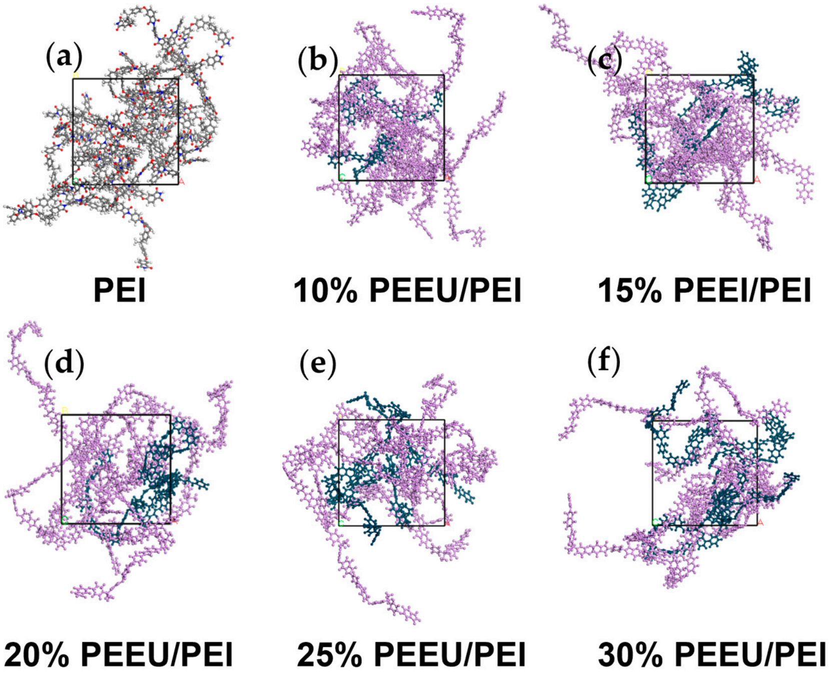

Figure S5. The stereochemical structures of PEI and PEI/PEEU films by DFT calculation.

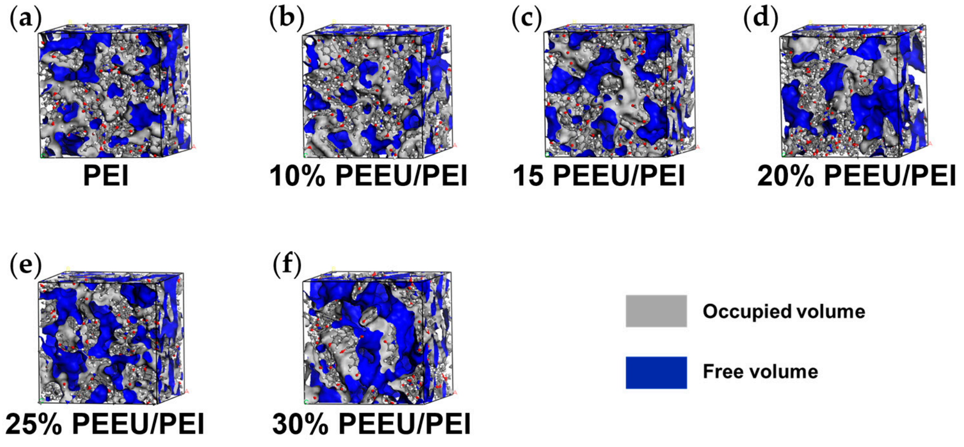

Figure S6. Free volume simulation.

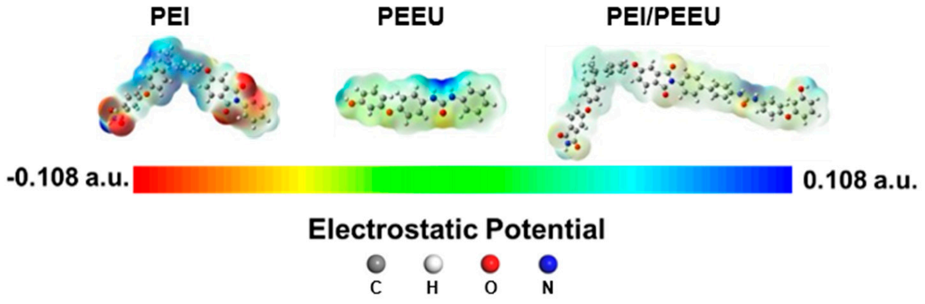

Figure S7. Three-dimensional electrostatic potential.

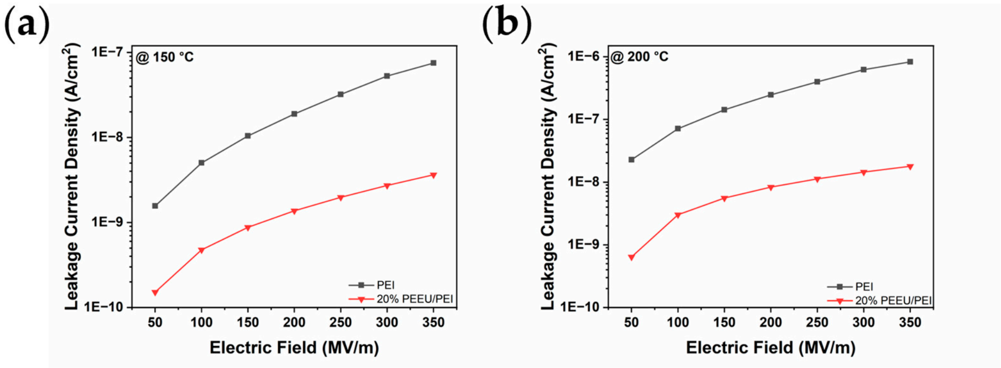

Figure S8. Leakage current density of PEI and 20% PEEU/PEI, **(a)** 150 °C, **(b)** 200 °C.

Dielectric materials store energy through electric displacement in response to an external electric field. The maximum polarization (P_m) is achieved as the electric field approaches the breakdown field (E_b). However, during the discharge process, the presence of hysteresis losses means only a portion of the stored polarization (energy) can be recovered, resulting in a remnant polarization (P_r) when the field is removed [3].

The discharged energy density (U_d) is represented by the dark green area of the loop and is calculated as:

$$U_d = \int_{P_r}^{P_m} E\mathrm{dP} \quad \text{(S1)}$$

The light green area represents the energy losses (U_l), which are due to hysteretic polarization switching or leakage current. The energy storage efficiency (η) is calculated by taking the ratio of the discharged energy to the total stored energy:

$$\eta = \frac{U_d}{U_d + U_l} \quad \text{(S2)}$$

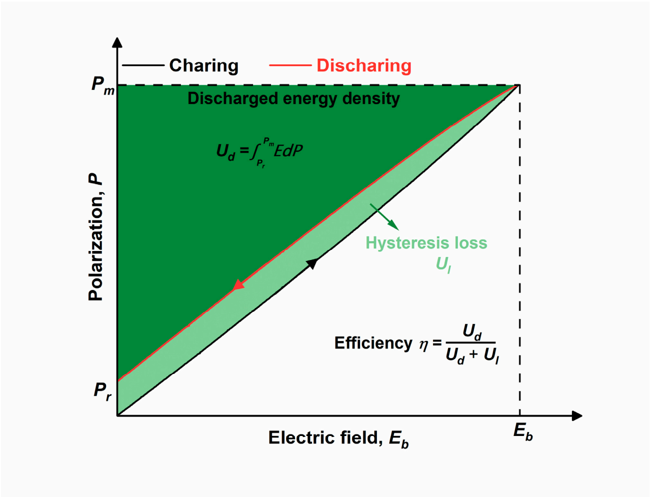

Figure S9. Schematic diagram of *P*-*E* loop.

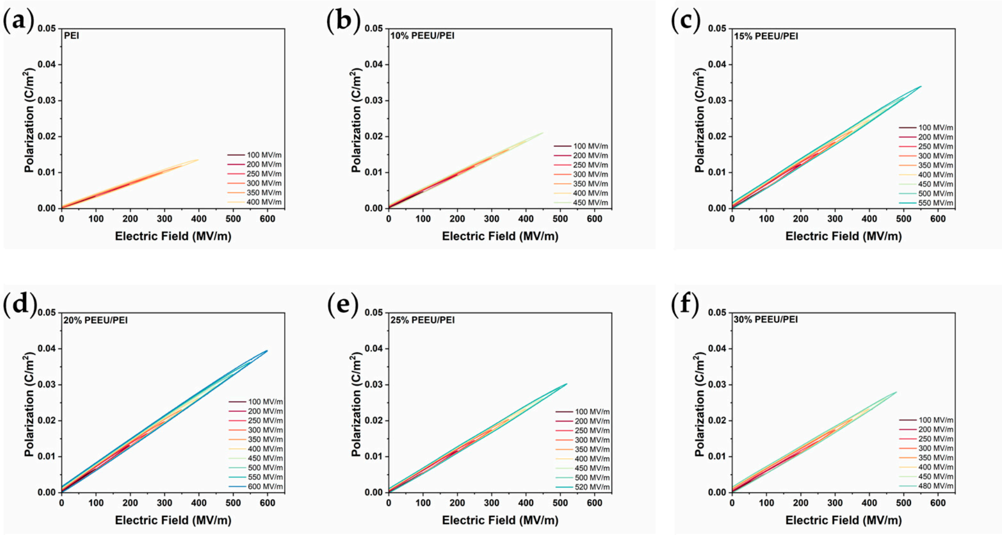

Figure S10. *P-E* loops of **(a)** PEI, **(b)** 10% PEEU/PEI, **(c)** 15% PEEU/PEI, **(d)** 20% PEEU/PEI, **(e)** 25% PEEU/PEI, **(f)** 30% PEEU/PEI, at 25 °C.

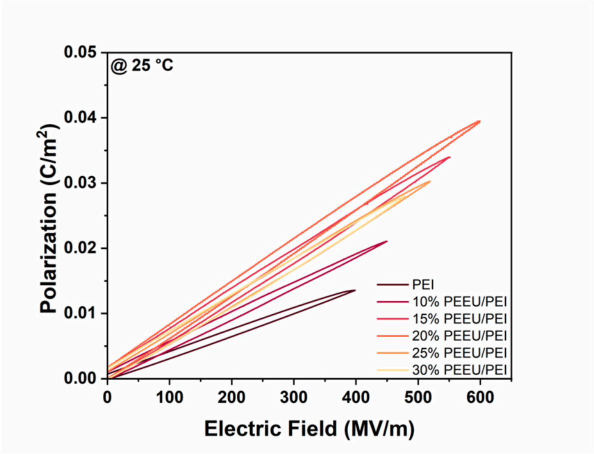

Figure S11. Comparison of *P-E* loop at 25 °C.

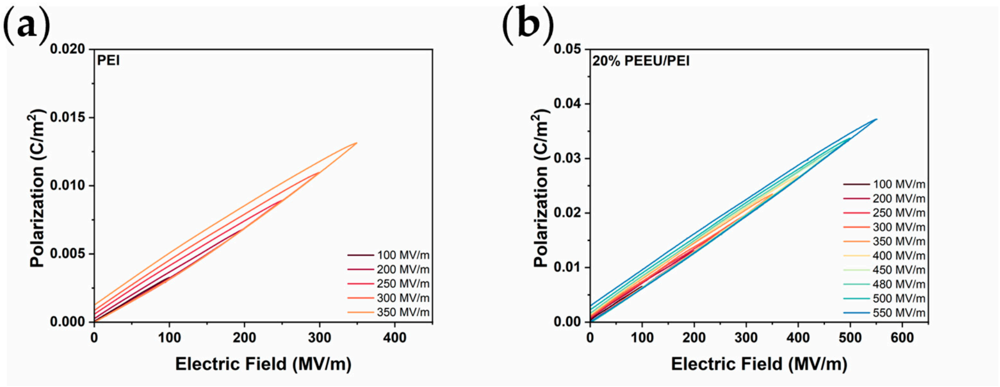

Figure S12. *P-E* loops of **(a)** PEI, **(b)** 20% PEEU/PEI at 150 °C.

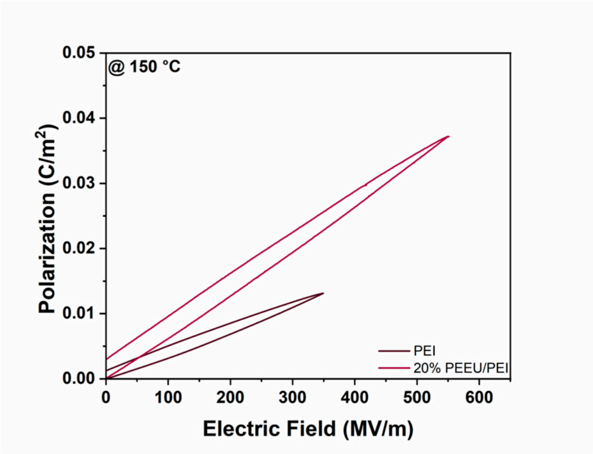

Figure S13. Comparison of *P-E* loop at 150 °C.

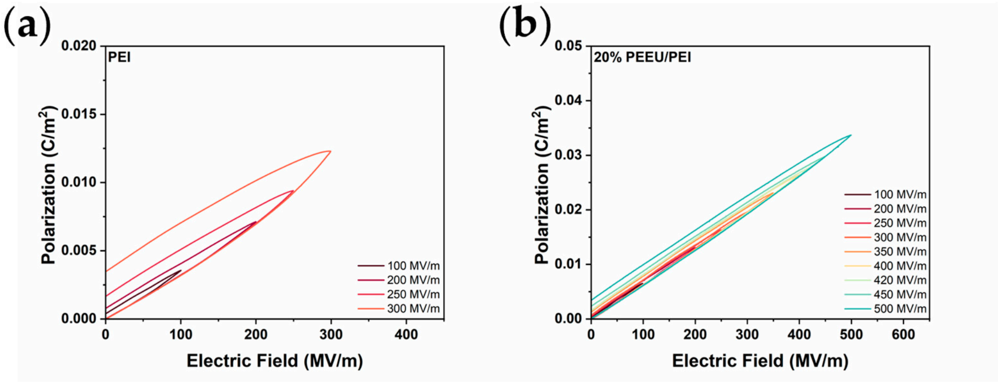

Figure S14. *P-E* loops of **(a)** PEI, **(b)** 20% PEEU/PEI at 200 °C.

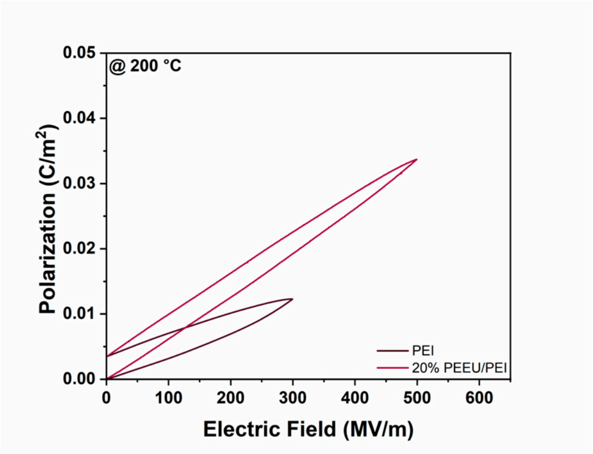

Figure S15. Comparison of *P-E* loop at 200 °C.

To ascertain the dielectrics' breakdown strength (E_b), a minimum of 10 points were chosen from various areas within each sample for E_b testing. The analysis of the results was conducted using the two-parameter Weibull statistic formula [4]:

$$P(E) = 1 - \exp\left[-\left(E / E_b\right)^{\beta}\right] \tag{S3}$$

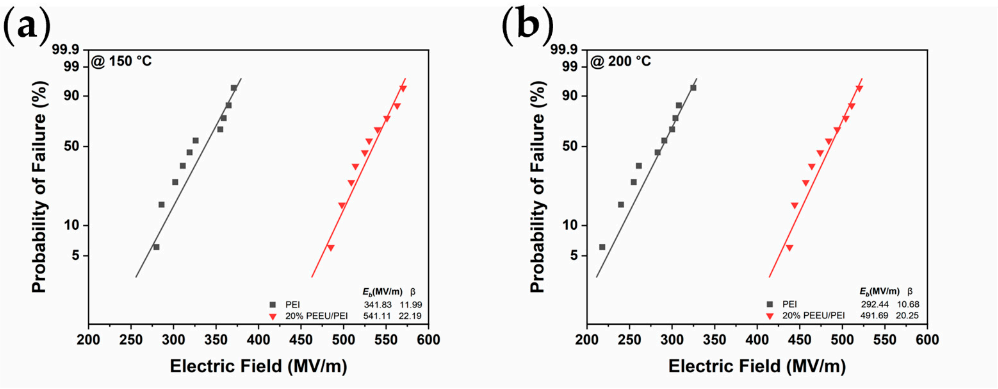

Figure S16. Weibull breakdown strength of PEI and 20% PEEU/PEI **(a)** 150 °C, **(b)** 200 °C.

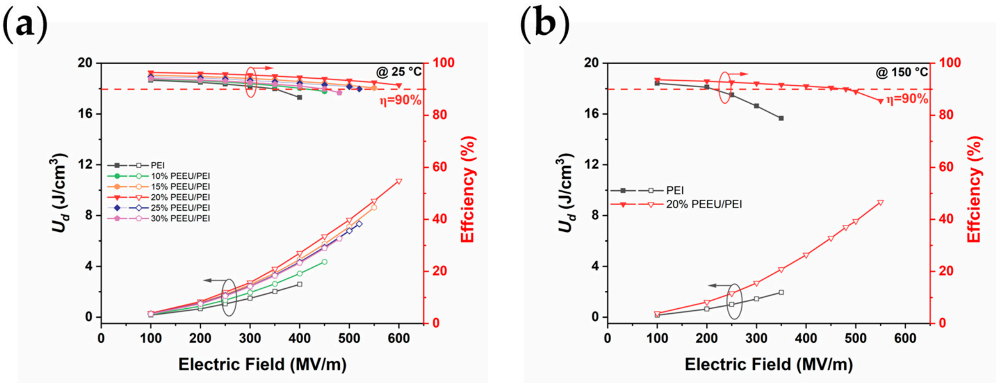

Figure S17. Comparison of discharged energy density and charge-discharge efficiency of PEI and 20% PEEU/PEI, **(a)** 25 °C, **(b)** 150 °C.

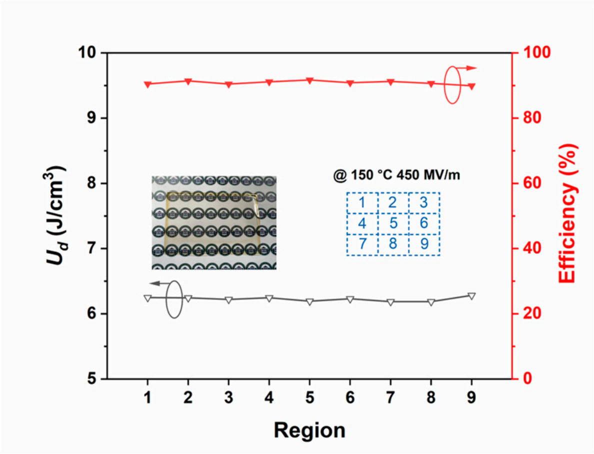

Figure S18. Energy storage performances of different regions in large scale 20% PEEU/PEI. The inset is the photograph of the 20% PEEU/PEI.

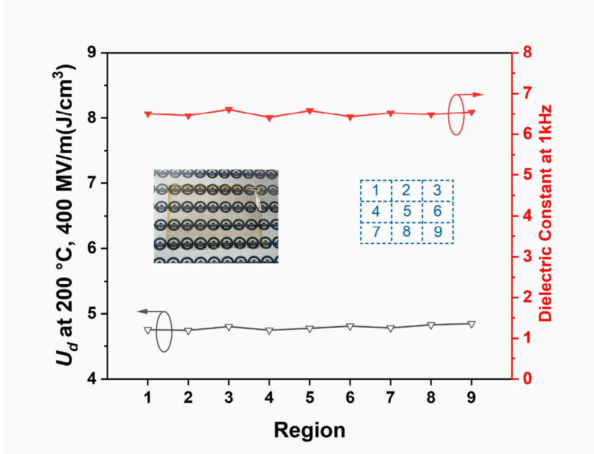

Figure S19. Energy storage and dielectric constant performances of different regions in large scale 20% PEEU/PEI. Standard deviations of U_d is 0.036. Standard deviations of K is 0.070.

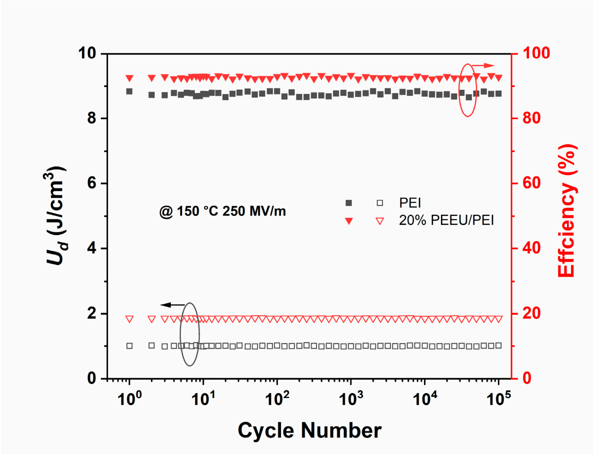

Figure S20. Cyclic performance at 150 °C, 250 MV/m.

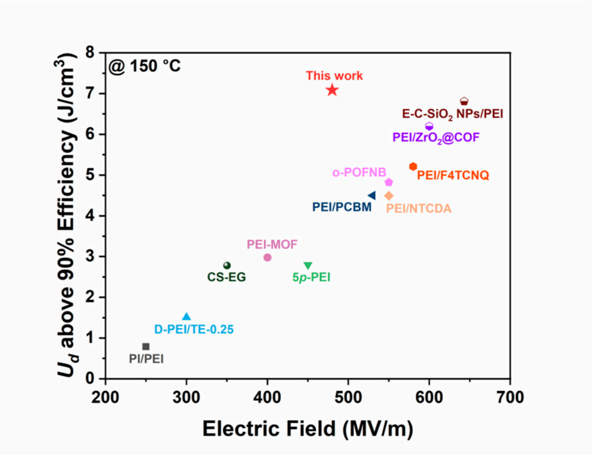

Figure S21. Maximum discharged energy density achieved above 90% efficiency at 150 °C of 20% PEEU/PEI and recently published high-temperature dielectric polymers [5-15].

Table S1. Dielectric metrics of PEI and PEEU/PEIs.

Polymers	K (1 kHz)	Dielectric Loss (1 kHz)	E_b (MV/m) at 25 °C	U_{dmax} (J/cm^3) at 25 °C	η (%)
PEI	3.20	0.0131	400	2.58	86.86
10% PEEU/PEI	4.61	0.0090	450	4.37	89.21
15% PEEU/PEI	5.79	0.0063	550	8.64	90.50
20% PEEU/PEI	6.51	0.0045	600	10.74	91.57
25% PEEU/PEI	5.50	0.0066	520	7.35	90.12
30% PEEU/PEI	4.99	0.0076	480	6.18	88.65

Table S2. Dielectric constant, Free volume fraction and Free Volume of PEI and PEEU/PEIs.

Polymers	*K* (1 kHz)	Free volume fraction (%)	Free Volume (A^3)
PEI	3.20	36.08	14536.09
10% PEEU/PEI	4.61	37.00	14784.04
15% PEEU/PEI	5.79	42.79	15620.44
20% PEEU/PEI	6.51	45.32	19905.95
25% PEEU/PEI	5.50	40.46	15093.00
30% PEEU/PEI	4.99	38.65	14916.69

Reference

1. Boshoman, S.; Fatoba, O.; Dada, O.; Jen, T. Transition metal oxide catalytic abilities for fuel cell applications: Density functional theory (DFT) studies. *Materials Today Communications* **2024**, *39*, 109125.
2. Wu, Q.; Deng, D.; Zhou, R.; Zhang, J.; Zou, W.; Liu, L.; Wu, S.; Lu, K.; Wei, Z. Modulation of donor alkyl terminal chains with the shifting branching point leads to the optimized morphology and efficient all-small-molecule organic solar cells. *ACS applied materials & interfaces* **2020**, *12*, 25100-25107.
3. Zhou, Y.; Wang, Q. Advanced polymer dielectrics for high temperature capacitive energy storage. *Journal of Applied Physics* **2020**, *127*.
4. Kim, H.; Shi, F. Thickness dependent dielectric strength of a low-permittivity dielectric film. *IEEE Transactions on Dielectrics and Electrical Insulation* **2002**, *8*, 248-252.
5. Xie, Z.; Le, K.; Li, H.; Pang, X.; Xu, T.; Altoé, V.; Klivansky, L.M.; Wang, Y.; Huang, Z.; Shelton, S.W. Interfacial engineering using covalent organic frameworks in polymer composites for high‐temperature electrostatic energy storage. *Advanced Functional Materials* **2024**, *34*, 2314910.
6. Wang, Z.; Zhao, Y.; Yang, M.; Yan, H.; Xu, C.; Tian, B.; Zhang, C.; Xie, Q.; Dang, Z.M. Surface Strengthening of Polymer Composite Dielectrics for Superior High‐Temperature Capacitive Energy Storage. *Advanced Energy Materials* **2025**, *15*, 2405411.
7. Deshmukh, A.A.; Wu, C.; Yassin, O.; Mishra, A.; Chen, L.; Alamri, A.; Li, Z.; Zhou, J.; Mutlu, Z.;

Sotzing, M. Flexible polyolefin dielectric by strategic design of organic modules for harsh condition electrification. *Energy & Environmental Science* **2022**, *15*, 1307-1314.

8. Zhang, B.; Chen, X.m.; Pan, Z.; Liu, P.; Mao, M.; Song, K.; Mao, Z.; Sun, R.; Wang, D.; Zhang, S. Superior high‐temperature energy density in molecular semiconductor/polymer all‐organic composites. *Advanced Functional Materials* **2023**, *33*, 2210050.
9. Zhang, Q.; Chen, X.; Zhang, B.; Zhang, T.; Lu, W.; Chen, Z.; Liu, Z.; Kim, S.H.; Donovan, B.; Warzoha, R.J. High-temperature polymers with record-high breakdown strength enabled by rationally designed chain-packing behavior in blends. *Matter* **2021**, *4*, 2448-2459.
10. Yuan, C.; Zhou, Y.; Zhu, Y.; Liang, J.; Wang, S.; Peng, S.; Li, Y.; Cheng, S.; Yang, M.; Hu, J. Polymer/molecular semiconductor all-organic composites for high-temperature dielectric energy storage. *Nature communications* **2020**, *11*, 3919.
11. Meng, Z.; Zhang, T.; Zhang, C.; Dang, Z.M.; Chi, Q. Optimizing energy storage performance in polymer dielectrics through dual strategies: constructing "peaked" barrieras and enhancing carrier scattering. *Advanced Functional Materials* **2024**, *34*, 2403402.
12. Pan, Z.; Li, L.; Wang, L.; Luo, G.; Xu, X.; Jin, F.; Dong, J.; Niu, Y.; Sun, L.; Guo, C. Tailoring poly (styrene‐co‐maleic anhydride) networks for all‐polymer dielectrics exhibiting ultrahigh energy density and charge–discharge efficiency at elevated temperatures. *Advanced Materials* **2023**, *35*, 2207580.
13. Dong, X.; Wang, Y.; Cao, Y.; Li, N.; Fu, J.; Yu, J.; Hu, Z. Enhanced high-temperature energy storage performance in all-organic dielectric films through synergistic crosslinking of chemical and physical interaction. *Chemical Engineering Journal* **2024**, *500*, 157312.
14. Wang, F.; Cai, J.; Yang, C.; Luo, H.; Li, X.; Hou, H.; Zou, G.; Zhang, D. Improved capacitive energy storage nanocomposites at high temperature utilizing ultralow loading of bimetallic MOF. *Small* **2023**, *19*, 2300510.
15. Alamri, A.; Wu, C.; Mishra, A.; Chen, L.; Li, Z.; Deshmukh, A.; Zhou, J.; Yassin, O.; Ramprasad, R.; Vashishta, P. Improving the rotational freedom of polyetherimide: enhancement of the dielectric properties of a commodity high-temperature polymer using a structural defect. *Chemistry of Materials* **2022**, *34*, 6553-6558.
